# *De novo* Assembly and Characterization of the Floral Transcriptomes of Two Varieties of *Melastoma malabathricum*

**DOI:** 10.3389/fgene.2019.00521

**Published:** 2019-06-19

**Authors:** Tao Zheng, Yihua Lin, Longping Wang, Qiujin Lin, Xiuxiang Lin, Zhendong Chen, Zhenyue Lin

**Affiliations:** ^1^Fujian Institute of Tropical Crops, Zhangzhou, China; ^2^Institute of Oceanography, Minjiang University, Fuzhou, China; ^3^College of Ocean and Earth Sciences, Xiamen University, Xiamen, China; ^4^Xiamen Forest Quarantine and Prevention Station, Xiamen Greening Administration Center, Xiamen, China

**Keywords:** *Melastoma malabathricum*, floral transcriptome, lineage-specific expression, flavonoid, floral ontogeny

## Abstract

*Melastoma malabathricum* is an important medicinal and landscape plant that is globally distributed in temperate and subtropical regions. However, available genomic information for the entire Melastomataceae family is notably limited. In view of the application potential of floral parts in secondary metabolite extraction, we characterized for the first time the floral transcriptomes of two key *M. malabathricum* varieties, purple variety and white variety. Our transcriptome assembly generated 52,498 and 49,380 unigenes with an N50 of 1,906 and 1,929 bases for the purple and white varieties, respectively. Comparative analysis of two transcriptomes demonstrated that they are highly similar but also highlighted genes that are presumably lineage specific, which explains the phenotypes of each variety. Additionally, a shared transcriptional signature across the floral developmental stages was identified in both *M. malabathricum* varieties; this signature included pathways related to secondary metabolite synthesis, plant hormone signaling and production, energy homeostasis and nutrient assimilation pathways, and cellular proliferation. The expression levels of flavonoid accumulation and candidate flavonoid biosynthesis-related genes in *M. malabathricum* flower development stages validated the transcriptome findings. The transcriptome data presented in this study will serve as a valuable resource for future work on the exploitation of *M. malabathricum* and other related species. The gene expression dynamics during flower development will facilitate the discovery of lineage-specific genes associated with phenotypic characteristics and will elucidate the mechanism of the ontogeny of individual flower types.

## Introduction

*Melastoma malabathricum* (Melastomataceae) is an important traditional medicinal and landscape shrub that is commonly found in tropical and temperate Southeast Asian countries (Sharma et al., 2001; Joffry et al., 2012; Kumar et al., 2013). The natural interspecific hybridization of *Melastoma* is relatively common because of the overlap in geographic distribution, flowering time, and shared pollinators, which commonly create parental, intermediate, and even novel traits (Liu et al., 2014). The genus *Melastoma* comprises at least 22 species, two subspecies, and three varieties that are classified by the color of flower petals (Meyer, 2001; Rajenderan, 2010). Accordingly, *M. malabathricum* consists of three different varieties, including large-, middle-, and small-sized flowers with dark purple, light pink, and white (the rare variety, namely, *M. malabathricum* var. *alba*) petals that have similar morphology and pigment during their early development (Susanti et al., 2007; Joffry et al., 2012). These varieties also share other important traits, such as flowering time and stem types; thus, they may be useful in breeding and developing hybrid lineages (Renner and Meyer, 2001). Recent studies revealed that many phytochemicals from flowers have pharmacological properties, such as antinociceptive, anti-inflammatory, wound-healing, antidiarrheal, cytotoxic, and antioxidant activities (Rajenderan, 2010; Joffry et al., 2012; Roslen et al., 2014).

*M. malabathricum* has also been considered a potential source of anthocyanin (Suan See et al., 2011), and these compounds often accumulate at high concentrations in the flower; thus, in the future, they could be used as a source for the pharmaceutical and health supplement industries. To date, more than 30 phytochemicals have been identified in *M. malabathricum* flower extracts; most of these constituents are generalized flavonoids, anthocyanins, and alkaloids (Rasmussen et al., 2005). In general, the synthesis of these compounds is often an integral part of floral developmental regulation, which is tightly linked to the process of petal pigmentation and cell expansion (Susanti et al., 2007). Considering the genetic properties and developmental prospects possessed by different lineages of plants, it is important to study floral developmental processes in different *M. malabathricum* varieties. Therefore, characterizing the transcriptome dynamics during flower development helps to elucidate the molecular basis responsible for regulating the important natural products and the agronomic qualities of specific lineages in this nonmodel plant.

In this study, we deeply sequenced transcriptomes across the floral developmental stages from young unopened flower buds to mature opened flowers in two *M. malabathricum* varieties, the purple and white varieties, which exhibit purple petals and white petals, respectively, to generate the first transcriptomic resource for *Melastoma*. Then, we assessed the differential expression among three floral developmental stages to elucidate the molecular events underlying floral ontogeny in shrubs.

## Materials and Methods

### Plant Materials and Sampling

**Figure 1:**
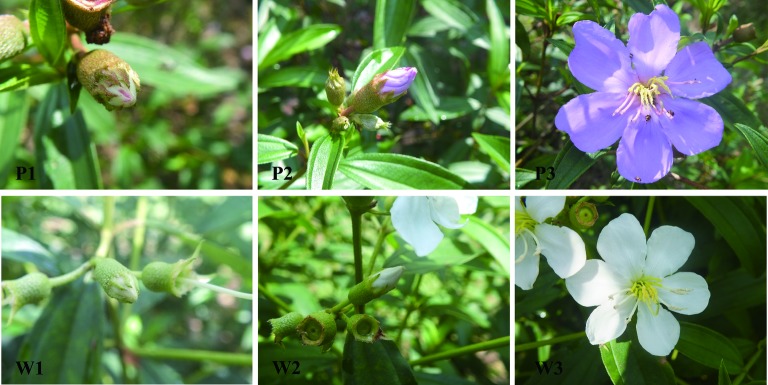
Transcriptome profiling during flower color development in the purple and white varieties of *M. malabathricum*. The successive stages of flower color development used in this study were unpigmented buds (stage 1), slightly pigmented buds (stage 2), and fully opened petals (stage 3). Stage 1 indicates that the flower buds have just emerged, and the last 10–12 days until stage 2 arise, wherein the buds’ length was equal to calyx. Stage 2 sustained 3–5 days until stage 3 appeared. The petals were fully opened in stage 3. P1–P3 represented stage 1 to stage 3 of the purple variety, and W1–W3 indicated stage 1 to stage 3 of the white variety.

The plant materials consisted of flowers from the purple and white varieties of *M. malabathricum* that had been grown under the same conditions in the peony base of the Guangzhou Institute of Landscape Gardening (Guangdong, China) in July 2016. Petal samples were collected at three flower color developmental stages for flavonoid content analysis and RNA extraction (Figure 1). Flowers were sampled from separate plants for each stage. The plants were at different stages, and the two varieties were randomized locations.

### RNA Extraction, Library Preparation, and Sequencing

Total RNA was isolated from each sample using a TRIzol® Reagent RNA Isolation Kit (Invitrogen, Grand Island, NY) following the manufacturer’s protocol. Three biological replicates of RNA were extracted for each sample. RNA degradation was detected on 1.2% agarose gels, and RNA purity was checked using a NanoPhotometer® spectrophotometer (IMPLEN, CA, USA). Approximately 5 μg of total RNA (pooled in equal amounts from three biological replicates) for each sample was used for library construction. mRNA was enriched by oligo(dT) beads, and sequencing libraries were created using an Illumina TruSeq™ RNA Sample Prep Kit (Illumina, SD, USA) following the standard high-throughput protocol. Sequencing was conducted on an Illumina HiSeq™ 4000 platform at Gene Denovo Biotechnology Co. (Guangzhou, China), and paired-end reads were generated.

### Transcriptome Assembly and Functional Annotation

Raw reads in the FASTQ format were first processed through in-house Perl scripts. Clean reads were obtained by removing reads containing adapters, reads containing poly-N, and low-quality reads from raw data. At the same time, the Q20, Q30, GC content, and sequence duplication level of the clean data were calculated. The remaining clean reads were assembled without a reference by Trinity for the two *M. malabathricum* varieties with a min_kmer_cov set to 2 by default, and all other parameters were set to the default (Grabherr et al., 2011). The obtained sequences were defined as unigenes. All of the unigenes were subjected to the Nr, SwissProt, COG, KEGG, and Pfam public databases to annotate gene functions. To identify the homologous genes of two *M. malabathricum* varieties by similarity searching with an *E* < 10^−5^ using BLASTn, the GATK3 software (McKenna et al., 2010) was used to perform SNP calling. Raw vcf files were filtered with the GATK standard filter method with default parameters.

### Differential Expression and Enrichment Analysis

High-quality clean reads were compared to *de novo* assemblies using Bowtie2, and then the RPKM (reads per kb per million read) values were calculated and normalized (Mortazavi et al., 2008). To identify DEGs across samples without biological replicates, for each sequenced library, the read counts were adjusted by edgeR package through one scaling normalized factor (Robinson et al., 2010). Differential expression analysis of any two samples was performed using the edgeR package. We identified genes with a log2 fold change ≥2 and a corrected *p* <0.01 in comparison as significant DE genes. We used the KOBAS software with a hypergeometric test and the Benjamini-Hochberg FDR correction to test the statistical enrichment of DE genes in KEGG pathways (Xie et al., 2011).

### Flavonoid Extraction and Quantification Analysis

Flavonoid content was determined according to commonly used spectrophotometric methods based on the formation of a complex flavonoid, aluminum, which has an absorption maximum at 510 nm (Pękal and Pyrzynska, 2014), and using quercetin as the internal standard. Briefly, the flower petal materials were dried to a specific weight, chopped very finely, and sieved through a plastic sieve (<0.5 mm). The dried material (1.0 g) was crushed by ultrasonication and extracted for 1 h with 2.5 ml of 60% (v/v) aqueous ethanol at 60°C. Then, the precipitate solution was separated by centrifugation, and pellets were discarded. One milliliter of the diluted supernatant sample was separately mixed with 1 ml of 2% aluminum chloride methanol solution (Djeridane et al., 2006). After incubation at room temperature for 15 min, the absorbance of the reaction mixture was measured at 510 nm with a Milton Roy 601 UV-vis spectrophotometer, and the flavonoid content was expressed in milligrams per gram of dry weight. All analyses were carried out with at least four replicates, and ANOVA was performed on the means of the flavonoid content. SPSS Statistics v. R23.0 (IBM) was used for data analysis with ANOVA (PROC ANOVA). Treatment means were separated at the 0.05 significance level by Tukey’s test.

### Quantitative PCR Analysis

To validate the transcriptome, RNA samples from three biological replicates of each stage were adjusted to 1 μg/μl with nuclease-free water. Two micrograms of total RNA was reverse transcribed in a 20-μl reaction volume using the Prime Script™ RT reagent Kit with gDNA Eraser (Takara, Dalian, China). Nine genes were chosen, and quantitative PCR was performed with an ABI Step-One plus Real-Time PCR system (Applied Biosystems, Canada). Each reaction was carried out in triplicate, and the primers used for qPCR were designed based on the transcriptome data except for actin and are listed in Supplementary Table S1. Relative expression levels of target genes were determined by the 2^−△△Ct^ method (Livak and Schmittgen, 2001). The correlation between the expression profiles of the selected genes measured by qPCR and RNA-seq was determined using the R package.

## Results

### Transcriptome Sequencing and Assembly

To profile the flower transcriptome of two *M. malabathricum* varieties with contrasting purple and white petals, we sequenced six RNA-seq libraries from three successive developmental stages of flowers, including young bud (stage 1), buds about to open (stage 2), and buds fully opened (stage 3) from the purple and white varieties of *M. malabathricum* (Figure 1). The early petal color of the purple variety is unpigmented, slightly pigmented, and completely turned to purple during stages 1–3. However, the petal color of the white variety in the three stages is white.

After adapter removal and quality trimming, we obtained a total of 195,675,054 reads for the purple variety and 180,277,772 reads for the white variety, for a total of 29 and 26 Gb of clean nucleotides, respectively. The percentages of clean reads among the raw tags (Q20) ranged from 97.41 to 97.65% (Table 1). We conducted *de novo* assembly of the transcriptomes of the two *M. malabathricum* varieties by Trinity (Grabherr et al., 2011) and obtained 52,498 and 49,380 unigenes for the purple and white varieties, respectively. The average unigene length and other characteristics of the assembly in the two *M. malabathricum* varieties were similar (Table 1 and Supplementary Figure S1). The sequencing coverage (estimated as the mean number of reads per unigene) was assessed as 3,169 for the purple variety and 3,052 for the white variety. BUSCO analysis (Simão et al., 2015) revealed a completeness score of approximately 70% for each assembly (Supplementary Figure S2). These results demonstrate that the assembled unigenes could be a useful source of relatively complete coding sequences for *M. malabathricum*.

**Table 1:** Summary of the sequencing data generated and mapped to the corresponding *M. malabathricum* variety transcriptome assembly.

Sample	Raw reads	Clean reads	Clean base (bp)	Total mapped reads (%)	GC (%)	Q20 (%)	Q30 (%)
P1	66879504	65081046	9728175931	54419101 (84.97)	49.72	97.65	94.32
P2	72984558	70689320	10571797430	59749469 (85.02)	49.97	97.41	93.85
P3	61664010	59904688	8950259129	51201673 (85.97)	50.43	97.53	94.09
Total/average (purple-variety)	201528072	195675054	29250232490	166351295 (85.26)	50.04	97.53	94.09
W1	66604434	64606226	9659650195	53660248 (83.48)	49.96	97.51	94.05
W2	60905058	58993728	8814083921	48911732 (83.58)	51.87	97.42	93.86
W3	58413404	56677818	8473748955	47550854 (84.41)	50.60	97.52	94.06
Total/average (white-variety)	185922896	180277772	26947483071	150693372 (83.85)	50.81	97.48	93.99

### Transcriptome Annotation

In total, 34,856 (67%) and 34,715 (70%) unigenes were successfully annotated with a significance threshold (*E* ≤ 1E−5) in at least one of the Nr, SwissProt, COG, and KEGG public databases for the purple and white varieties, respectively, and the results are summarized in Table 2. Interestingly, for *M. malabathricum*, the top hit species was *Eucalyptus grandis*, or flooded gum, followed by *Theobroma cacao* and then *Cephalotus follicularis* (Supplementary Figure S3A), suggesting that it is more closely related to flooded gum. Annotated unigenes were categorized into 25 functional groups according to the COG classification (Supplementary Figure S3B). The distribution of COG categories in both varieties is largely similar; only some categories showed slight differences between them. For example, the purple variety showed a considerably higher number of genes than the white variety, including general function prediction, signal transduction mechanisms, and transcription. In turn, two pathways were particularly abundant in the white variety, “energy production and conversion” and “translation, and ribosomal structure, and biogenesis.” These annotated transcriptomes can facilitate the discovery of lineage-specific biological functions of *M. malabathricum* genes. Additionally, the transcripts were annotated against all plant transcription factors (TFs), resulting in 1,597 and 1,603 annotated transcripts for the purple and white varieties, respectively. Comparative analysis of the TF gene family compositions revealed significant differences between the two varieties (Supplementary Figure S3C, Supplementary Table S2). This exceptionally large and diverse inventory of TFs may play an important role in floral ontogeny in shrubs.

**Table 2:** Summary of the *M. malabathricum* transcriptome assembly.

	Purple variety	White variety
**Transcriptome assembly**		
Total unique sequences	52,498	49,380
Total base pair (Mb)	55.5	52.18
Average (bp)	1,057	1,056
N50 size (bp)	1,906	1,929
Minimum sequence length (bp)	201	201
Maximum sequence length (bp)	16,634	16,125
GC%	47.23	47.86
**Transcriptome annotation**		
Nr	34,605	32,752
Swissprot	25,826	27,091
KOG	21,448	20,084
Kegg	14,058	13,428
Annotation genes	34,856	34,715
Without annotation gene	17,642	14,665

### Comparative Analysis of the *M. malabathricum* Transcripts and SNPs

**Figure 2:**
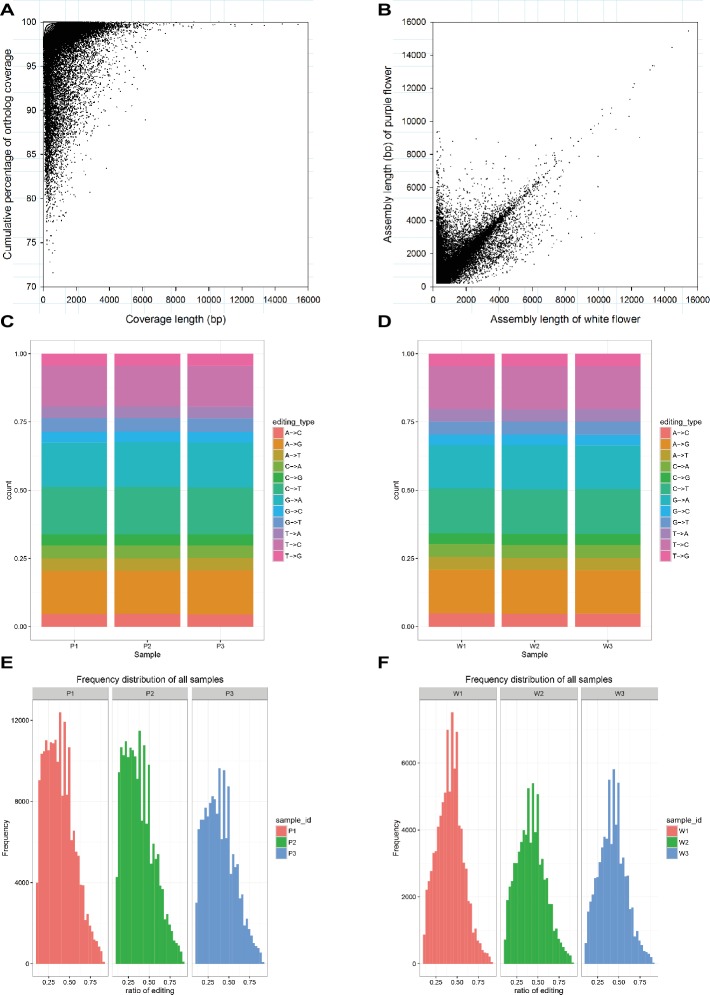
Characteristics of the transcriptome assembly in *M. malabathricum*. Coverage and assembly length in orthologous unigenes **(A,B)**. Each dot represents a pair of orthologs. Distribution of the different SNP types in the purple **(C)** and white **(D)** varieties. Frequency of the different ratio of RNA editing in the purple **(E)** and white **(F)** varieties.

As mentioned earlier, the raw data characteristics and assembly, as well as the annotation characteristics, were largely similar for the purple and white varieties. We further investigated these transcriptomes for homology based on the corresponding BLASTn hits that aligned to one another (*E* < 1E−5). In total, 41,026 unigenes covered more than 70% of their best BLAST hit and showed that there is a strong correlation between the assembly length of predicted unigenes that are orthologous in the purple variety and the white variety (Figures 2A,B). To make a comparison, SNPs in the assembled *M. malabathricum* transcriptome were also examined. As a result, the distribution of each SNP type was similar in the two varieties (Figures 2C,D). Our results also showed similarity in the frequency distribution of RNA editing in three stages of flowering for either variety, indicating that this is a general pattern of RNA editing across the floral developmental stages. However, there is a clear distinction in the frequency distribution for RNA editing between the two varieties, and the editing efficiency was significantly higher in the purple variety compared to the white variety (Figures 2E,F), which may be directly linked to the expressed genes of the contrasting genotypes. Taken together, comparative analysis of two transcriptomes demonstrated their overall similarity but also revealed a presumably lineage-specific transcriptomic signature.

### Expression Dynamics of the Orthologous Genes

**Figure 3:**
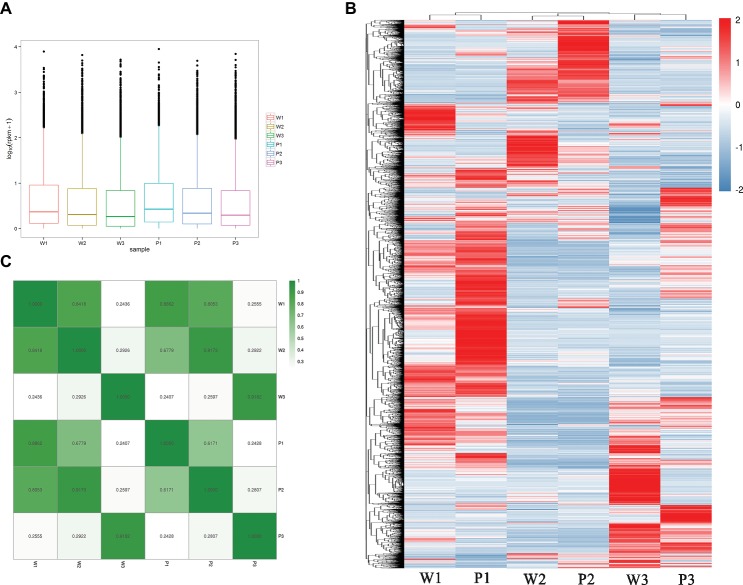
Transcriptome dynamics during the various stages of flower development. **(A)** Distributions of the expression levels of all unigenes. **(B)** Heatmap comparing scaled expression values for *M. malabathricum* purple and white varieties across developmental stages. The blue bands indicate low gene expression levels; the red bands indicate high gene expression levels. **(C)** Pairwise correlations of gene expression between any stages of flower development in two *M. malabathricum* varieties are shown. The differentially expressed genes were identified by comparing the RPKM with |log2 fold change| > 2.0 and FDR-adjusted *p* ≤ 0.01.

To estimate the expression levels, high-quality reads from each sample were mapped to the corresponding *M. malabathricum* variety transcriptome assembly. The overall transcriptional activity of all the genes was normalized, and abundance was estimated based on RPKM values. In general, the distributions of the expression levels of most of the genes were similar for the two *M. malabathricum* varieties in the corresponding stages. Higher expression levels were observed in flower development stage 1 followed by stage 2, and the lowest expression was observed in stage 3 (Figure 3A). To observe the orthologous gene expression patterns, a heatmap that represents the hierarchical clustering of homologous transcripts between different developmental stages revealed that samples of the two *M. malabathricum* varieties at the same stage were grouped together and that stage 1 was strongly differentiated from stage 2 and stage 3 (Figure 3B). Similarly, despite the small relative distinction between varieties at the same stage, specific gene expression levels for individual plant varieties were clearly presented in this study. As expected, the correlation of the same stage between the purple variety and the white variety was high (*r* from 0.886 to 0.918) compared with the correlation of the different stages from the purple or white variety (Figure 3C). These data revealed that many shared transcriptional signatures across floral developmental stages were activated in both *M. malabathricum* varieties.

### Metabolic Pathway Enrichment Analysis

**Figure 4:**
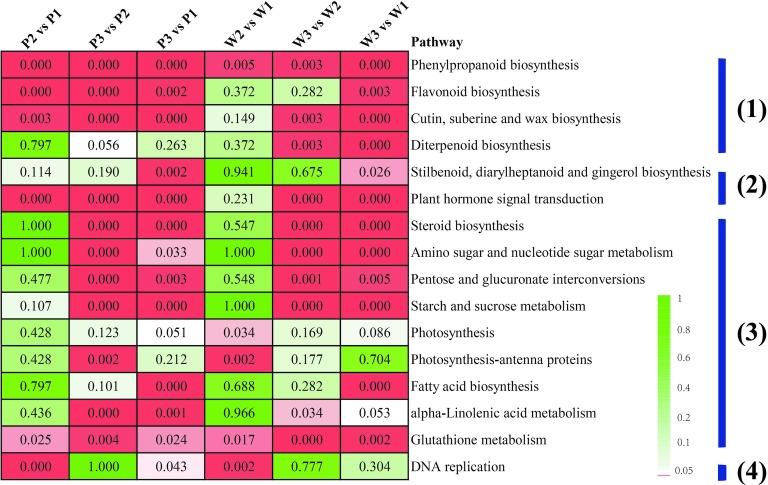
Enriched KEGG pathways for differentially expressed genes between any two *M. malabathricum* varieties. The number represents the FDR value. The red box indicates a pathway that was significantly enriched (the FDR value is <0.05).

Pairwise comparisons among the preceding stages, with respect to each *M. malabathricum* variety, were performed to gain insight into the gene expression regulation of flower development. A false discovery rate (FDR) cutoff of 0.01 and log2 fold change of ±2 were set as the thresholds for putative differentially expressed genes (DEGs). The number of DEGs at the different developmental stages is shown in Supplementary Table S3. The enrichment analysis with KEGG terms (FDR < 0.05) in both DEG sets is largely similar, with only a small number of categories being unique for either variety. The four major metabolic pathway categories included (1) secondary metabolite synthesis, such as phenylpropanoid, flavonoid, cutin, wax, and diterpenoid biosynthetic pathways; (2) plant hormone signaling and production; (3) energy homeostasis and nutrient assimilation pathways, including the energy substrate biosynthesis and interconversions (such as pentose, glucuronate, starch and sucrose, fatty acids, and steroid biosynthesis), amino acid, nucleotide, and glutathione metabolism and photosynthesis; and (4) cellular proliferation, which appeared significantly overrepresented in flower development in the two varieties of *M. malabathricum* (Figure 4). It is also worth mentioning that most of the pathways were found to be significantly enriched in the last half of floral development, from stages 2 to 3 (stage 3 vs. stage 2). In contrast, in the initial pigmented period of stages 1 and 2 (stage 2 vs. stage 1), more KEGG enrichment categories were only marginally significant or not significant (FDR > 0.05), except for a limited number of pathways, such as phenylpropanoid biosynthesis, glutathione metabolism, and DNA replication.

Although many of the enriched KEGG terms were common, several of them were unique in different sets of genes. For example, phenylpropanoid biosynthesis was the most represented in a linage-specific expression manner in the purple variety compared to the white variety over flower developmental stages 1–3. Furthermore, three overrepresented pathways related to flavonoid biosynthesis, diterpenoid biosynthesis, and plant hormone signal transduction were also identified as the specific category for the initial flower pigmentation developmental process (P1 vs. W1). These results suggest that there is a potentially unique constitutive metabolism in initial flower development in each variety, possibly representing differences in lineage-specific molecular expression-derived morphological characteristics.

### Assessment of Overrepresented Metabolic Pathway Accuracy

**Figure 5:**
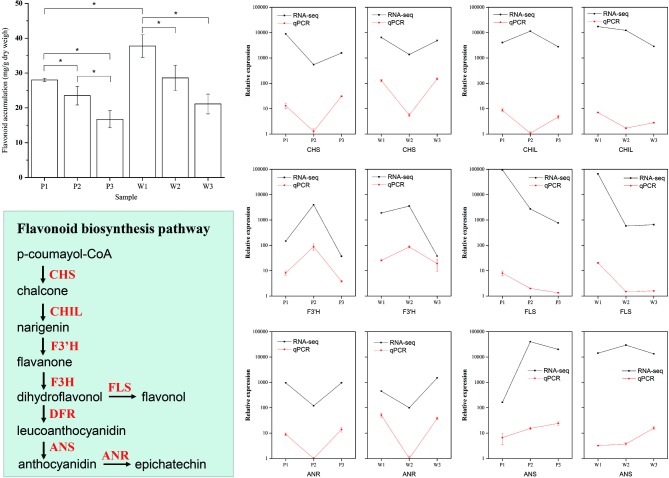
Validation of the flavonoid biosynthetic pathways in differentially expressed genes during flower development obtained by RNA-seq. Flavonoid abundance levels during flower color development. The amounts of flavonoids in petals at the three developmental stages and in the purple (P) and white (W) varieties determined by spectrophotometric analysis. Values indicate the average of four replicates for each sample. Verification of RNA-seq results by qPCR. The qPCR data are presented as mean ± SD (*n*=9), and the error bars represent the SD (*p < 0.05).

To assess the accuracy of the pathway enrichment from the DEG analysis obtained using RNA-seq data, we quantified flavonoid production in all three flower development stages in both *M. malabathricum* varieties to validate whether flavonoid biosynthesis was overexpressed (Figure 5). The results of the variance analysis showed that the accumulation of flavonoids drastically decreased with the flower developmental stages, particularly in the purple variety. The flavonoid content was significantly higher in white petals in stage 1 relative to that in purple petals (*p* < 0.01). Furthermore, we performed qPCR analysis for six selected genes associated with flavonoid synthesis, and the qPCR results were consistent with the RNA-seq data (*r*^2^ = 0.83). The temporal expression patterns were highly similar between the *M. malabathricum* purple and white varieties, with the exception of the magnitude of differences; for example, the magnitude of variation of F3’H and ANS was markedly increased in the purple variety. The final biosynthetic steps for flavonoids that were catalyzed by FLS showed strong downregulation in the developing phase, which had an overall effect of decreasing the pool of FLS transcripts that, in turn, could limit the metabolic flux of flavonoids. The enzymes of ANS that catalyze the late steps of anthocyanin biosynthesis had significantly higher transcript induction for the purple variety development at both stage 2 and stage 3 relative to that observed for the white variety; low expression of the ANS gene resulted in little accumulation of anthocyanins in the white variety, which supported the hypothesis regarding its importance in the regulation of plant colors (Shimizu et al., 2011). These analyses suggest that there is a temporal regulation of flavonoid biosynthesis with flower development in both varieties, which agrees with the overrepresentation of the flavonoid biosynthesis pathway in the transcriptome analysis. Thus, flavonoid biosynthesis is important to the regulation of flower color development in *M. malabathricum*.

## Discussion

To date, limited genomic data have been available for *Melastoma* plants, with only the chloroplast genome being available in NCBI genomes. This lack of data has greatly restricted molecular studies of a valuable medicinal and botanical resource. Fortunately, the transcriptome sequence of *M. malabathricum,* which does not have a reference genome sequence, is an effective tool for identifying the spatiotemporal differential expression of genes (Lin et al., 2017). In this study, considering the potential for floral applications, we sequenced two floral transcriptomes corresponding to three stages of flowering in two varieties, white and purple, of *M. malabathricum*. We performed the independent assembly of the transcriptome of the two *M. malabathricum* varieties because in an independent transcriptome assembly, the information concerning transcript variants (with SNPs, indels, or alternative splicing) between the two plants can be detected. We assembled ~50 Mb to the transcriptome sequence from both *M. malabathricum* varieties. The data represent an important genetic resource of nearly 50,000 transcripts, most of which are homologous across varieties, but many of which are presumably lineage specific. Moreover, the temporal variation in gene expression in this study revealed potential key pathways and processes that underlie the progression of flower development and will contribute to functional studies of the molecular regulation of flower development in *M. malabathricum* and other related nonmodel species.

*Melastoma* is among the most abundant and diversified groups of plants throughout the tropics, but their intraspecific relationships and genetic connectivity within species are poorly understood. The resultant phylogenetic construction revealed that Melastomataceae belong to Myrtales, where it is sister to the small CAP clade (Crypteroniaceae, Alzateaceae, and Penaeaceae), which, taken together, form a clade sister to Myrtaceae + Vochysiaceae (Reginato et al., 2016). In both *M. malabathricum* varieties, the species that provided most of the top BLAST hits is another Myrtales plant, *E. grandis* (approximately 10,000 *Melastoma* genes had the strongest similarity to *Eucalyptus* genes). The *Eucalyptus* nuclear genome has been sequenced (Myburg et al., 2014). Two varieties of the purple and white petal types are known in *M. malabathricum*, and they are very similar but differ in color. This type of floral color change is significantly correlated with the RNA level transcriptional regulations (Bradley et al., 2017). In contrast, a high frequency of RNA editing is detected across the floral developmental stages in both varieties. Significant differentiation in RNA level editing efficiency is found between the color types in varieties, which may play a crucial role in guiding the phenotypic differentiation to specific lineage fates.

Flowering represents the developmental transition from the vegetative to the reproductive stage in most angiosperm plants (Samach et al., 2000; Valverde et al., 2004). There was a strong trend indicating that the floral development stages of each variety of *M. malabathricum* were correlated with gene expression. A shared transcriptional signature across the developmental stages in two pigment types of *M. malabathricum* reveals that the floral development evolution likely originated from an ancestral genetic signature in which separate components overlapped (Chanderbali et al., 2010). In the early stages of flower color development (stages 1 and 2), gene expression was biased toward terms related to secondary metabolite synthesis [one secondary metabolite group is polyphenols, including flavonoids and phenylpropanoid (Scalbert et al., 2005), and other large-molecular-weight substances, such as diterpenoids, wax and cutin], cellular proliferation, and plant hormone production. Our results are concordant with the theory that the majority of petal pigmentation occurs during the rapid-growth phase of flower development, which is tightly linked to the process of cell expansion (Sun et al., 2015) and gives rise to the coordinated expression of multiple related genes that are involved in cell wall synthesis, such as wax and cutin biosynthesis (Fich et al., 2016). Previous studies have proposed that flower pigmentation is due to the accumulation of flavonoids and anthocyanins, which are also influenced by vacuolar pH, co-pigmentation, and the shape of the petal cells (Mol et al., 1998). Flavonoids are synthesized in plants *via* the flavonoid branch of the phenylpropanoid pathway (Buer et al., 2010). Previous studies also suggested that the enzyme complexes in the endoplasmic reticulum were sited for phenylpropanoid and flavonoid metabolism in petal tissue (Hrazdina and Wagner, 1985) and that coordinated enzyme and gene expression occurs within the same cell. During flower growth, there is an increased expression of genes involved in diterpenoid and flavone biosynthesis, which was largely congruent with previous observations in *Rosmarinus officinalis*. Del Baño suggests that the distribution of these compounds in the first stages of flower growth is due to *in situ* biosynthesis but that in the last stages, transport is increased (del Baño et al., 2003). Flavonoids have important roles in influencing the transport and production of plant hormones and auxin, and these pathways could also be categorized in color development according to flavonoid roles in many facets of plant physiology (Peer and Murphy, 2007).

Many abovementioned development processes are also highly active in later color developmental stages (stages 2 and 3), where many pathway terms related to energy storage and nutrient assimilation metabolism were also enriched. The flowering process is characterized by rapid cell division followed by cell expansion and accumulation of storage products, mainly in the form of starch or proteins (Ruan et al., 2010). Degradation of polysaccharides, proteins, lipids, and nucleic acids results in mobilization of sugars and nitrogenous compounds through the phloem to other plant parts before causing petal senescence (van Doorn, 2004). Photosynthesis is one of the most important pathways in carbon accumulation; carbon not only functions as the major energy building block source but also plays crucial roles in signaling molecules, in which partitioning in plants is regulated at the molecular level during development (Zhang et al., 2010). Weiss showed that photoperiod promotes pigmentation (i.e., anthocyanin) at even later stages of corolla development (Weiss, 2000). Additionally, glutathione pathways are required for postembryonic meristematic activity, and they regulate a common cell cycle regulator in the formation of the flower (Reichheld et al., 2007). Recently, two genes (DcGSTF1 and DcGSTF2) with homology to glutathione *S*-transferase (GST) were identified from carnation and showed high levels of transcription at the late stages of petal development, which might be responsible for intense color in carnation flowers (Sasaki et al., 2012).

Although the characterized functional properties of the genetic composition and differential expression gene sets in the two *M. malabathricum* varieties were similar overall, our results also highlighted the importance of lineage-specific transcripts involved in the regulation of flower development. The differential analysis of the transcripts assembled, the annotation characteristics, and the expression dynamics between the two *M. malabathricum* plants may be related to the color traits in these two lineages. For example, in this investigation, TF abundance of gene family composition showed significant differences in the purple variety compared to the white variety. Floral transcriptional regulators are broadly influenced by the focal to peripheral organ categories imparting intergrading morphologies and pigments across floral organs (Chanderbali et al., 2010). Among the various TF families, MYB, ERF, bHLH, NAC, WRKY, and GRAS will be differently abundant between the purple and the white variety, which might impact lineage-specific transcriptome dynamics and result in phenotypic qualities to some extent in *M. malabathricum* under the dosage effect. Previous studies have revealed that the zinc finger family and ERF proteins are involved in development, which induces spontaneous somatic embryo formation (Boutilier et al., 2002). The members of the MYB and bHLH families have also been reported to be involved in the biosynthesis and/or transport regulation of flavonoids, anthocyanins, and phenylpropanoids in other plant species (Laitinen et al., 2008; Czemmel et al., 2012). Taken together, this study’s results indicate that *M. malabathricum* flowering temporal gene expression dynamics may similarly have dual roles in the flowering time inherent regulative signature and the genetic characteristics of different lineages. Our results open avenues for future research in the field.

## Conclusions

This study generated two complete floral transcriptome assemblies in the nonmodel shrubby plant species *M. malabathricum.* These transcriptomic data, as well as their functional annotations, provide ample resources for genetics and breeding studies in *M. malabathricum*. Moreover, the differential gene expression dynamics during flower development will facilitate the discovery of lineage-specific genes associated with lineage-specific phenotypic characteristics and will elucidate the mechanism of the ontogeny of individual flower types.

## Author Contributions

ZC and ZL conceived the study. YL, TZ, and ZL performed the flavonoid analysis and wrote the manuscript. LW, XL, and QL contributed to the sample collection and phenotypic analyses. ZL conducted the bioinformatics analysis and revised the manuscript. All authors read and approved the final manuscript.

### Conflict of Interest Statement

The authors declare that the research was conducted in the absence of any commercial or financial relationships that could be construed as a potential conflict of interest.
