# Supplementary material for: De novo Assembly and Characterization of the Floral Transcriptomes of Two Varieties of Melastoma malabathricum
Source: Front Genet. 2019 Jun 19;10:521. doi: 10.3389/fgene.2019.00521 (PMC6594232; doi:10.3389/fgene.2019.00521)
Supplement: Supplementary file 1 [file Data_Sheet_1.docx]

Table S1. Primers used for qPCR.

| Primer name | Sequence(5'-3') |
| --- | --- |
| CHS-F1 | GCTTCGGGACCTCAACCA |
| CHS-R1 | TCACCAACAGCGAGCACAA |
| F3'H-F1 | TGCTCTCAAGGTTGGATCTG |
| F3'H-R1 | CTTTCGCTCATCTCTGTTTGG |
| ANS-F2 | AGCGACACCAGGATTTTCGT |
| ANS-R2 | TAAGACCCCGAGCGACTACA |
| ANR-F3 | CGTCCCCTCGAGCTTGTTAG |
| ANR-R3 | GCTGCGACTACGTCTTCCAT |
| CHIL-F2 | CGAGAGATTTCTGCGGGTGA |
| CHIL-R2 | CGGTGTACTTGCCGATGGAT |
| FLS-F1 | TCCGTGCCTTCTCTTTGTTC |
| FLS-R1 | TCAAAGACAGGAAGTGGTACG |
| actin-F1 | AGTTGTTGTTCCGCTTTCCTC |
| actin-R1 | TTTTATCCTCCTCACCCTCCC |

Table S2. Annotated transcription factor number in public databases.

| Annotated transcription factors | white-variety | purple-variety |
| --- | --- | --- |
| MYB_related | 127 | 138 |
| ERF | 137 | 129 |
| bHLH | 124 | 117 |
| NAC | 93 | 98 |
| C2H2 | 86 | 81 |
| WRKY | 90 | 81 |
| MYB | 77 | 80 |
| GRAS | 84 | 73 |
| bZIP | 77 | 71 |
| G2-like | 57 | 58 |
| Dof | 52 | 48 |
| HD-ZIP | 45 | 45 |
| C3H | 48 | 45 |
| B3 | 33 | 44 |
| GATA | 34 | 38 |
| LBD | 38 | 36 |
| HSF | 33 | 33 |
| Trihelix | 30 | 32 |
| SBP | 26 | 31 |
| TCP | 28 | 24 |
| TALE | 17 | 22 |
| M-type | 21 | 21 |
| ARF | 17 | 19 |
| MIKC | 18 | 18 |
| HB-other | 13 | 17 |
| FAR1 | 16 | 17 |
| ZF-HD | 15 | 16 |
| YABBY | 7 | 12 |
| NF-YA | 11 | 11 |
| NF-YC | 12 | 11 |
| GRF | 9 | 10 |
| AP2 | 7 | 9 |
| DBB | 7 | 8 |
| LSD | 9 | 8 |
| NF-YB | 13 | 8 |
| BES1 | 12 | 8 |
| GeBP | 9 | 8 |
| CO-like | 6 | 7 |
| CPP | 6 | 7 |
| WOX | 7 | 6 |
| EIL | 5 | 6 |
| ARR-B | 7 | 5 |
| Nin-like | 4 | 5 |
| SRS | 4 | 5 |
| CAMTA | 3 | 4 |
| E2F/DP | 6 | 4 |
| BBR-BPC | 6 | 4 |
| RAV | 4 | 4 |
| NF-X1 | 2 | 3 |
| Whirly | 2 | 3 |
| VOZ | 3 | 3 |
| HRT-like | 1 | 2 |
| S1Fa-like | 2 | 1 |
| HB-PHD | 2 | 1 |
| STAT | 1 | 1 |

Table S3. List of differentially expressed genes in each comparison.

| Comparison | UP | Down |
| --- | --- | --- |
| P2 vs P1 | 6252 | 3258 |
| P3 vs P2 | 7437 | 4739 |
| P3 vs P1 | 10846 | 4533 |
| W2 vs W1 | 6211 | 3185 |
| W3 vs W2 | 7972 | 5400 |
| W3 vs W1 | 9209 | 3757 |
| P1 vs W1 | 2152 | 3936 |
| P2 vs W2 | 3566 | 5377 |
| P3 vs W3 | 1266 | 2852 |
