# Supplementary figures and images for: De novo Assembly and Characterization of the Floral Transcriptomes of Two Varieties of Melastoma malabathricum
Source: Front Genet. 2019 Jun 19;10:521. doi: 10.3389/fgene.2019.00521 (PMC6594232; doi:10.3389/fgene.2019.00521)

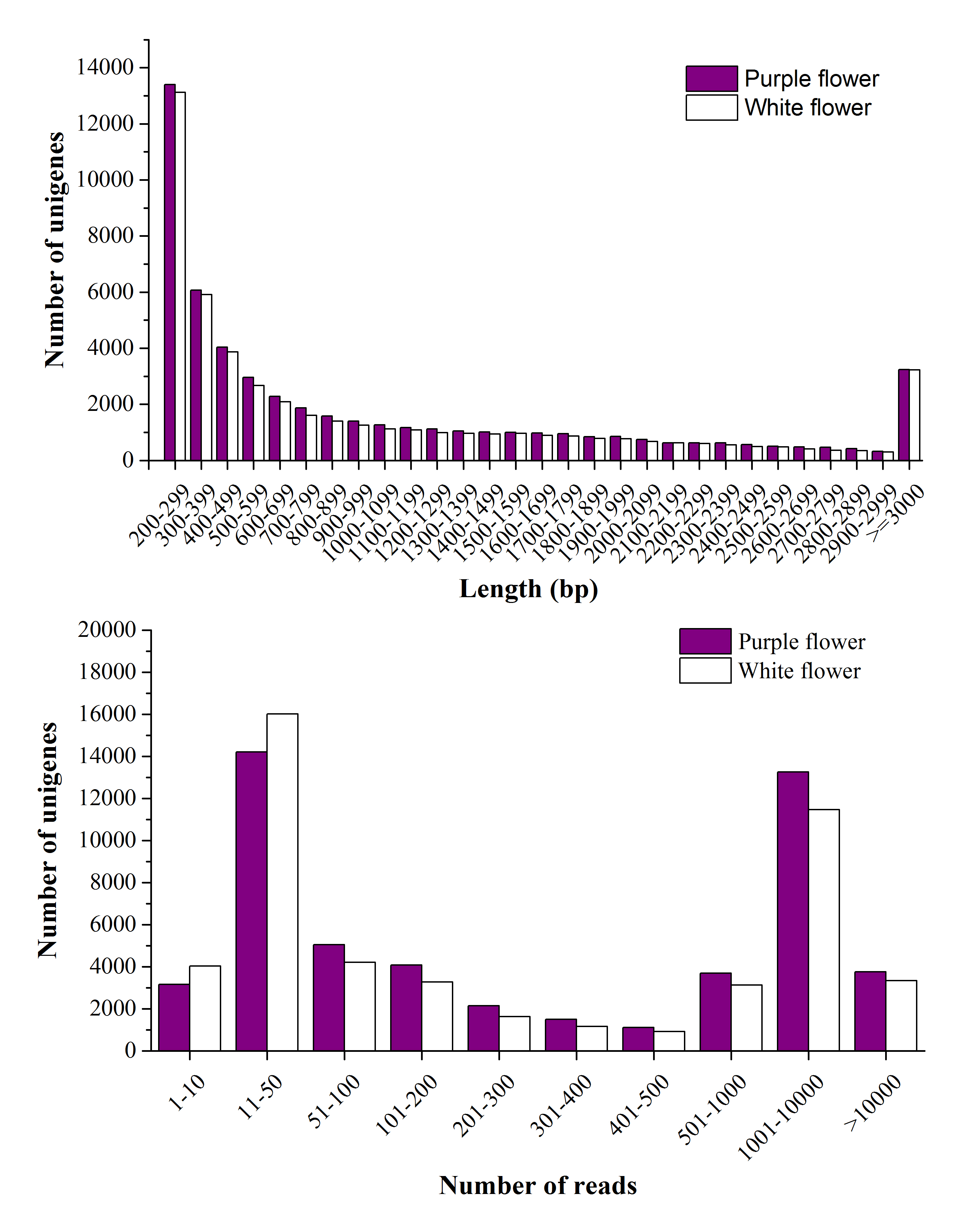

Supplement: Supplementary file 2 [file Image_1.TIF]

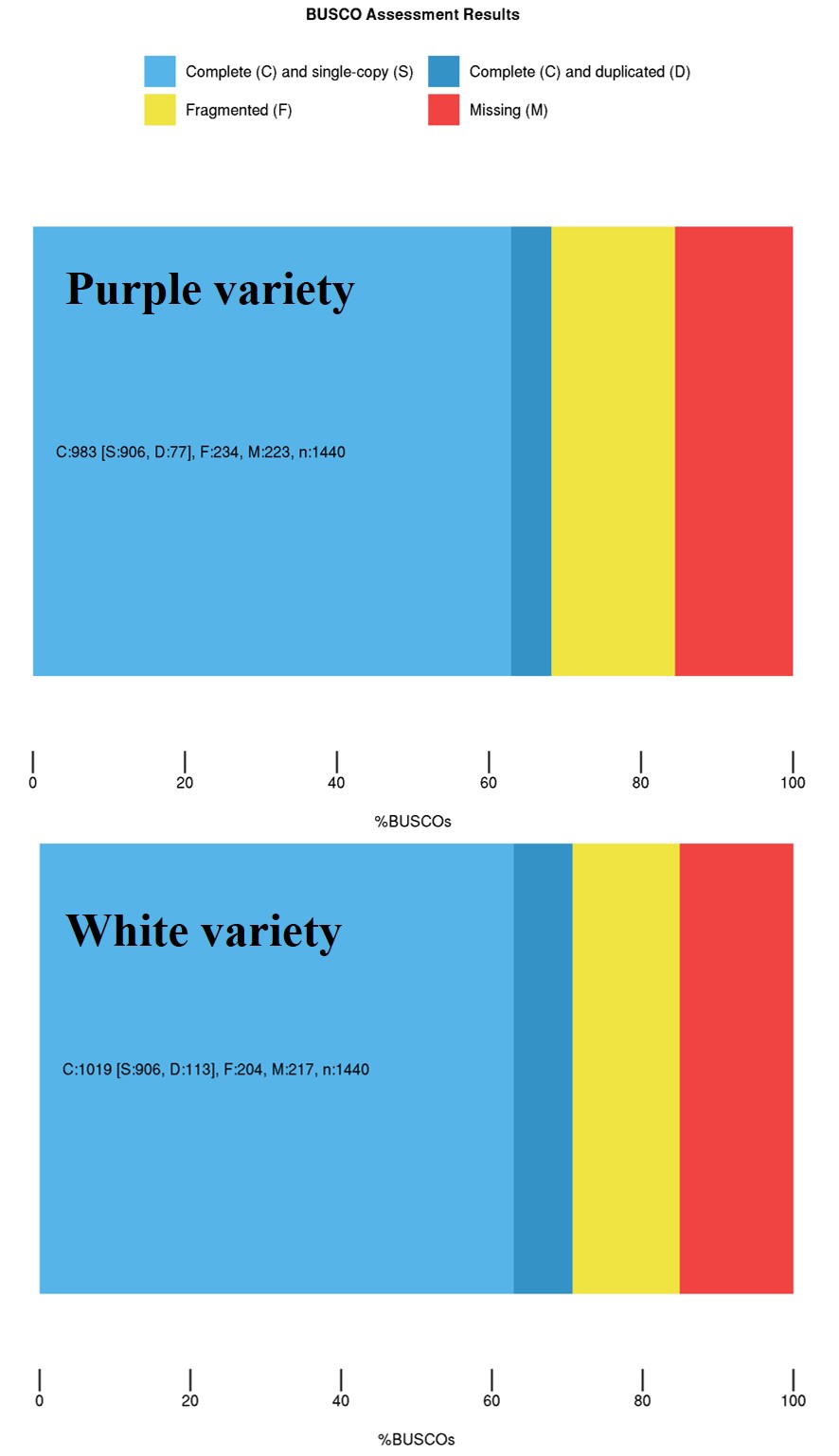

Supplement: Supplementary file 3 [file Image_2.JPEG]

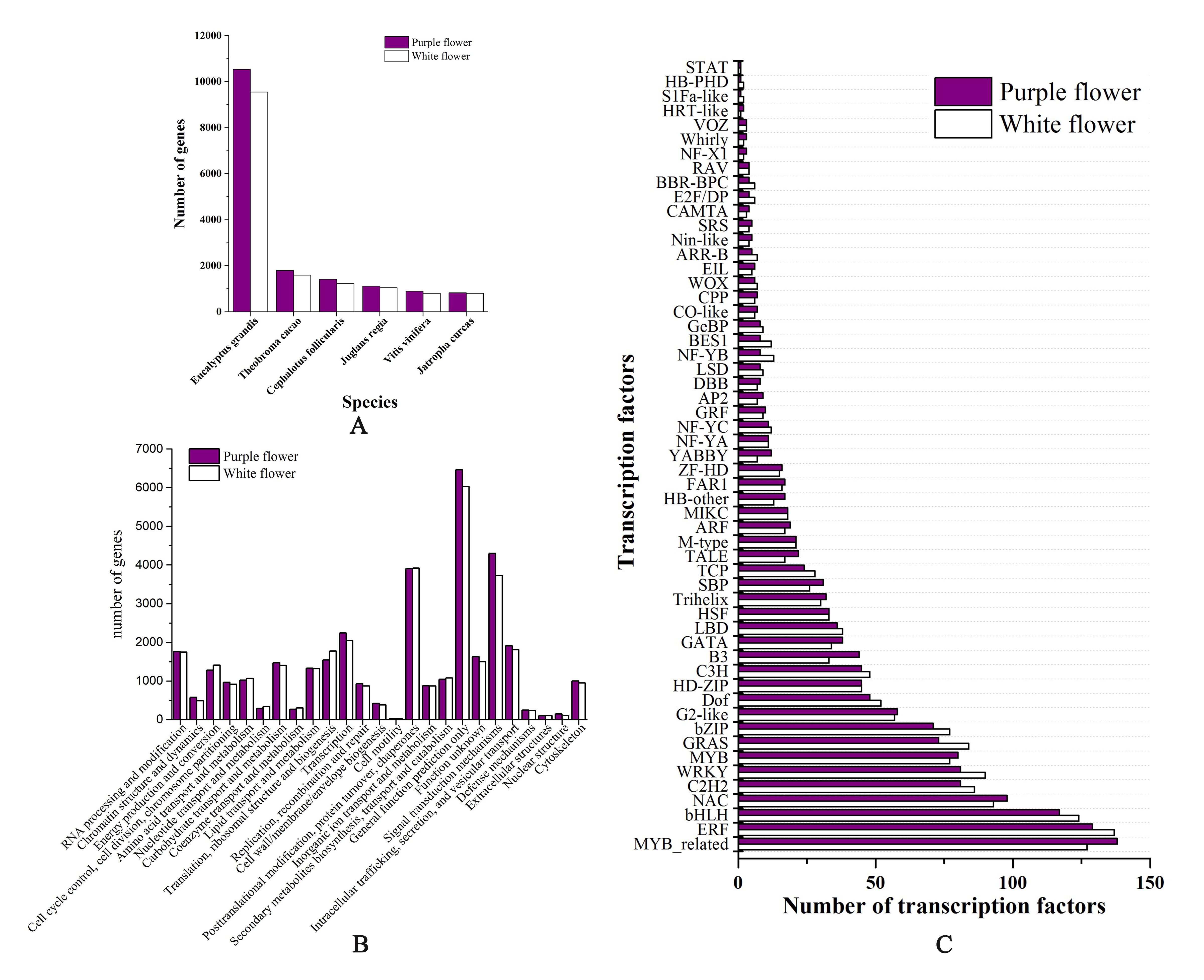

Supplement: Supplementary file 4 [file Image_3.TIF]
